# Sexual function is impaired in women and men with pulmonary hypertension

**DOI:** 10.1007/s00392-023-02214-3

**Published:** 2023-04-30

**Authors:** Paul M. Hendriks, Diederik P. Staal, Hester Pastoor, Corine I. A. Kolpa, Annemien E. van den Bosch, Marco C. Post, Karin A. Boomars

**Affiliations:** 1 Department of Respiratory Medicine, Erasmus MC, University Medical Center Rotterdam, Doctor Molewaterplein 40, 3015 GD Rotterdam, The Netherlands; 2 Department of Cardiology, Erasmus MC, University Medical Center Rotterdam, Rotterdam, The Netherlands; 3 Department of Cardiology, St. Antonius Hospital, Nieuwegein, The Netherlands; 4 Department of Obstetrics and Gynaecology, Division of Reproductive Endocrinology and Infertility, Erasmus MC, University Medical Center Rotterdam, Rotterdam, The Netherlands; 5 Department of Cardiology, Utrecht University Medical Center, Utrecht, The Netherlands

**Keywords:** Sexuality, Sexual dysfunction, Quality of life, Pulmonary hypertension, Pulmonary, Arterial hypertension

## Abstract

**Background:**

Sexual health related quality of life (SHRQoL) is an important pillar of health related quality of life (HRQoL). The aim of this study was to investigate sexual functioning in men and women with pulmonary hypertension (PH).

**Methods and results:**

In this cross-sectional study, a total of 78 patients were included, 49 were diagnosed with pulmonary arterial hypertension and 29 with chronic thromboembolic pulmonary hypertension (median age 53 [IQR: 46–67 years], 66.7% female). All patients completed SHRQoL questionnaires; for women: ASEX, FSFI, and FSDS and for men: ASEX and IIEF. A PH-specific SHRQoL questionnaire was created based on 4 semi-structured interviews to investigate PH-specific barriers in sexuality. More than half of the patients experienced symptoms during sexual activity, mainly dyspnea (52.6%) and palpitations (32.1%). Sexual dysfunction was present, according to the FSFI-questionnaire, in 63.0% of women. All of the men experienced at least mild dysfunction in one of the domains of the IIEF and erectile dysfunction was present in 48.0%. Sexual dysfunction occurred more often in both men and women with PH than in the general population. PAH-specific medication was not associated with sexual dysfunction, nor was subcutaneous or intravenous pump therapy (OR 1.14, 95%-CI: 0.75–1.73). Diuretics were associated with sexual dysfunction in women (OR 4.01, 95%-CI: 1.04–15.41). Of all patients committed in a relationship, 69.0% would like to discuss sexuality with their healthcare provider.

**Conclusion:**

This study showed a high prevalence of sexual dysfunction in men and women with PH. It is important for healthcare providers to discuss sexuality with patients.

**Graphical abstract:**

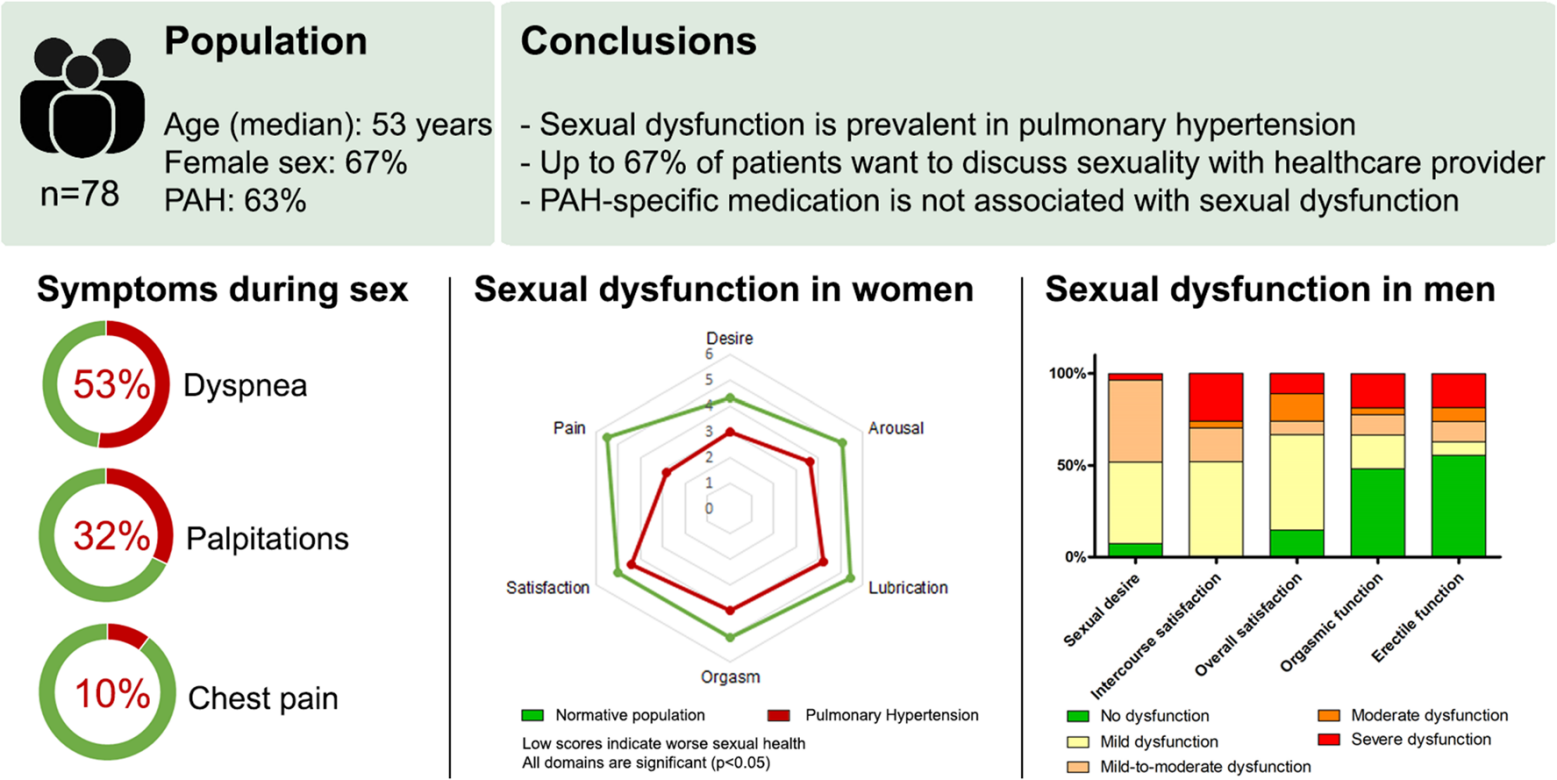

## Introduction

Pulmonary hypertension (PH) is a progressive disease affecting the pulmonary vasculature resulting in increased pulmonary vascular resistance and pulmonary arterial pressures. It is associated with high morbidity and mortality [1, 2]. Symptoms include progressive (exertional) dyspnea, fatigue, chest discomfort, palpitations, and cyanosis. Patients with PH are not only affected by physical symptoms, but also show decreased mental health, vitality and emotional and social functioning [3, 4]. Up to 80% of patients feel that family, friends, and colleagues do not understand their disease and its implications [5]. These factors contribute to a severely impaired health related quality of life (HRQoL) in patients with PH [3, 6, 7].

Sexual functioning is an important pillar of HRQoL and poor health, especially in chronic diseases, is accompanied by lower sexual satisfaction [8, 9]. Sexuality is a biopsychosocial phenomenon influenced by physical, psychological, and sociological factors. In PH, sexual health related quality of life (SHRQoL) is affected by multiple disease specific factors. First, patients experience worsening of cardiopulmonary symptoms during exertion. This could lead to discomfort or fear of symptoms during sexual activities. Second, impaired emotional and social functioning can result in sexual impairment. This can be aggravated by the feeling of patients that family or partners do not understand their disease. Patients experience low self-esteem and an impaired body image, especially when pump therapy is involved. These two factors are known to play a major role in sexual function [10, 11]. Additionally, women in their reproductive age could experience fear of pregnancy as pregnancy is highly discouraged in women with PH.

Not only that, patients might experience difficulties in engaging in sexual activities. Nearly three-quarters of caregivers report a decrease in sexual activity following the diagnosis of PH [12]. Furthermore, 23% feels less close to their spouse and 18% saw their spouse more as a patient they had to care for than as a partner [12]. More than 95% of PH patients report difficulties expressing themselves in general, but also to share their experience with PH specialists or nurses [5]. We expect that this percentage will even be higher regarding sexual information as this is known to be considered a taboo or difficult to mention for both patient and caregiver [13, 14].

It is known that sexual functioning is impaired in patients with chronic diseases and cardiopulmonary conditions including chronic heart failure, coronary heart disease, adult congenital heart disease, interstitial lung disease, diabetes mellitus and systemic sclerosis [15–21]. The evidence regarding sexual functioning in PH remains very limited. Only two previous studies described impaired sexual functioning in women with PAH and to our best knowledge, no study previously described sexual functioning in men with PH [22, 23]. The aim of this study was to investigate sexual functioning in men and women with PH.

## Study design and methods

### Patients and study design

In this cross-sectional study, patients diagnosed with pulmonary arterial hypertension (PAH) and chronic thromboembolic pulmonary hypertension (CTEPH) were approached for inclusion in two PH expertise centers in The Netherlands between March 2021 and October 2022. Patients were eligible when the diagnosis was confirmed by right heart catheterization according to the contemporaneous ESC/ERS guidelines of 2015 [24]. We excluded patients who were < 18 or > 80 years old, who underwent PEA and had no residual PH or who were unable to read or understand the Dutch questionnaires or the informed consent form. The study protocol was approved by the medical ethical committee of both participating centers (MEC-2020-0709, Z21.045) and written informed consent was provided by all participants. This study was endorsed by the Dutch national patient community for pulmonary hypertension.

### Patient assessment

Before the start of inclusion, four patients with different sex, disease etiology and therapy were asked to participate in a semi-structured individual interview regarding SHRQoL performed by a respiratory physician and nurse specialist. The structure of the interview was determined by a pulmonary physician, nurse specialist and sexologist. The interview was constructed to elicit (1) sexual desire; (2) psychosocial factors influencing sexuality; (3) physical symptoms during sexual activity; (4) self-esteem and body-image and (5) the need to discuss sexuality with a healthcare professional. Based on these interviews, we created a 10-item Dutch questionnaire, the Sexual health in Pulmonary Hypertension assessment (SePHia questionnaire). This assesses sexual health and problems in patients with PH and their wish to discuss sexuality with their healthcare providers.

All patients visited the outpatient clinic and underwent physical examination by a pulmonary physician or cardiologist, 6 min-walking test, and venous blood sampling for NT-proBNP. Participants were provided with questionnaires about general HRQoL and SHRQoL. General HRQoL was evaluated using the EmPHasis-10 questionnaire. It evaluates PH-specific symptoms based on 10 questions that are rated on a 5-point Likert scale. Higher scores represent worse HRQoL.

The Arizona Sexual Experience Scale (ASEX) assesses SHRQoL in both women and men. It consists of 5 questions that are rated on a 6-point Likert-scale (maximum score 30; lower scores indicate worse SHRQoL) [25]. Sexual dysfunction is defined as a score < 11. Each question assesses one of the following domains: sexual drive, arousal, erection/lubrication, orgasm and orgasmic satisfaction.

The Female Sexual Function Index (FSFI) evaluates sexual desire and functioning in females. It consists of 19 questions addressing six domains: desire, subjective arousal, lubrication, orgasm, satisfaction and pain. Questions are scored on either a 0-to-5 or 1-to-5 Likert scale. Low scores indicate worse sexual function and scores below 26.55 indicate sexual dysfunction. The Female Sexual Distress Scale (FSDS) measures sexual distress during sexual intercourse [26]. It consists of 12 questions scoring from 0-to-4 with higher scores indicating worse SHRQoL (maximum score 48). Based on both the FSFI and FSDS, sexual dysfunction can be defined as FSDS ≥ 11 and FSFI ≤ 26.55 [27].

The International Index of Erectile Function (IIEF) scores sexual health in males based on 15 questions (score range 0–5; higher scores indicating better sexual function). It addresses orgasmic function, sexual desire, intercourse satisfaction, overall satisfaction and erectile function.

The EmPHasis-10, ASEX, FSFI, FSDS and IIEF questionnaires are validated for external use. Normative values were extracted from the validation studies of the individual questionnaires and the general sexual functioning report of the Dutch Rutgers’ foundation [25, 26, 28–30]. The population used by the Dutch Rutger’s foundation is a random sample of the Dutch population consisting of 17.248 individuals. Forty-nine percent was male, 39% had higher education, 15.2% was single and 10–13% had a non-Western migration background [28].

### Statistical analysis

Continuous variables are presented as mean ± standard deviation or median (interquartile range) depending on their distribution. Categorical variables are presented as counts (percentage). Subgroup differences for continuous variables were analyzed using an unpaired *t* test or Wilcoxon one sample test depending on normality. The chi-square test or Fisher’s exact test was used to compare categorical variables.

Multivariable logistic regression with adjustment for age and sex was performed to assess the relation between sexual dysfunction and clinical characteristics & medication use. For logistic regression, we chose to define sexual dysfunction according to the ASEX questionnaire as this questionnaire applies to both men and women. Statistical analysis was performed using SPSS (IBM Corp. Released 2017, IBM SPSS Statistics for Windows, Version 25.0. Armonk, NY: IBM Corp.). A two-sided *p* value of < 0.05 was considered significant.

## Results

**Fig. 1:**
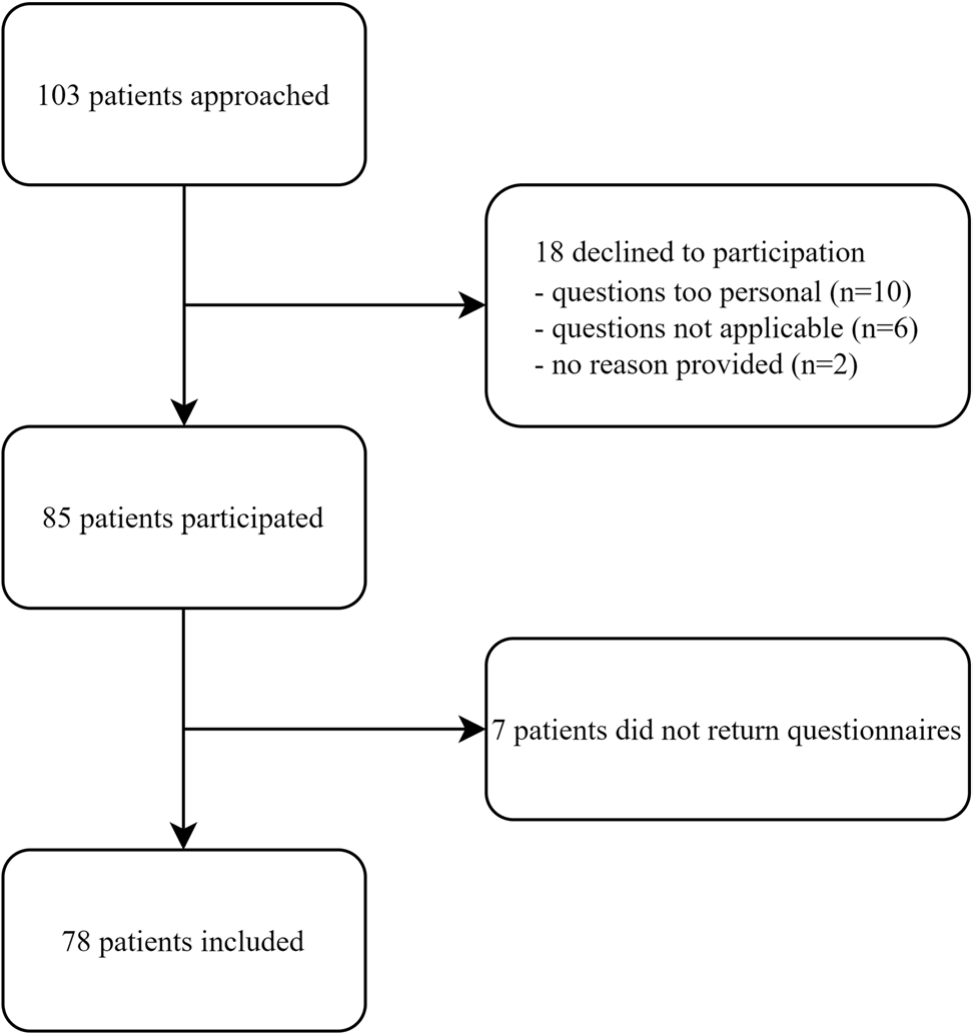
Flowchart of inclusion and response

A total of 103 patients were approached for inclusion of which 85 wanted to participate in the study (Fig. 1). Seven patients who were initially willing to participate did not return the questionnaires (response rate 91.8%). A total of 78 patients were finally included in the study with a median age of 53 (46–67) years and 62.8% was female (Table 1). Forty-nine patients (61.2%) were diagnosed with PAH and 29 (37.2%) with CTEPH. Patients with CTEPH were older at baseline and had a higher BMI. There were no other demographic differences between PAH and CTEPH. Among all patients, 77.3% was in a committed relationship.

**Table 1:** Baseline characteristics

	Total (*n* = 78)	PAH (*n* = 49)	CTEPH (*n* = 29)	*p* value
Demographic characteristics				
Age, years	53 (46–67)	49 (39–58)	67 (53–74)	< 0.001
Sex, female (%)	52 (66.7)	35 (71.4)	17 (58.6)	0.246
BMI, kg/m^2^	27.4 (22.7–30.7)	26.6 (22.0–29.5)	28.7 (25.4–35.8)	0.005
PAH-subtype				
Idiopathic PAH (%)	17 (34.7)	17 (34.7)	–	−
Hereditary PAH (%)	9 (18.4)	9 (18.4)	–	−
Connective tissue disease (%)	7 (14.3)	7 (14.3)	–	−
Congenital heart disease (%)	7 (14.3)	7 (14.3)	–	−
Portopulmonary hypertension (%)	4 (8.2)	4 (8.2)	–	−
Other (%)	5 (11.9)	5 (11.9)	–	−
Comorbidities				
Arterial hypertension (%)	15 (19.5)	9 (18.4)	6 (20.7)	0.771
COPD (%)	5 (6.5)	1 (2.0)	4 (13.8)	0.056
Coronary artery disease (%)	3 (3.9)	1 (2.0)	2 (6.9)	0.550
Diabetes mellitus (%)	9 (11.7)	6 (12.2)	3 (10.3)	0.576
6MWD, m	471 ± 132	498 ± 115	425 ± 148	0.344
NT-proBNP, pg/mL	169 (76–477)	161 (76–525)	178 (76–419)	0.934
NYHA class				0.176
I (%)	12 (15.4)	8 (16.3)	4 (13.8)	
II (%)	44 (56.4)	25 (51.0)	19 (65.5)	
III (%)	15 (19.2)	9 (18.4)	6 (20.7)	
IV (%)	7 (9.0)	7 (14.3)	0 (-)	
In a committed relationship (%)	58 (77.3)	39 (81.3)	19 (70.4)	0.280
Same sex relationship (%)	5 (6.3)	3 (6.1)	2 (6.5)	0.827
EmPHasis-10	19 (10–28)	20 (11–31)	17 (8–24)	0.327
Medication use				
Medication				
PDE5-inhibitor (%)	61 (78.2)	47 (95.9)	14 (48.3)	< 0.001
ET-1 antagonist (%)	66 (84.6)	44 (89.8)	22 (75.9)	0.099
Riociguat (%)	9 (11.5)	0 (–)	9 (31.0)	< 0.001
Selexipag (%)	13 (16.7)	12 (24.5)	1 (3.4)	0.013
Pump therapy (%)	17 (21.8)	16 (22.7)	1 (3.4)	0.052
Intravenous (%)	9 (52.9)	8 (50.0)	1 (100)	–
Subcutaneous (%)	4 (23.6)	4 (25.0)	0 (–)	–
Lenus Pro © (%)	5 (29.5)	4 (25.0)	0 (–)	–
PH-specific combination therapy				< 0.001
No PH-specific therapy (%)	3 (3.8)	0 (–)	3 (10.3)	
Monotherapy (%)	11 (14.1)	4 (8.2)	7 (24.1)	
Dual therapy (%)	37 (47.4)	20 (40.8)	17 (58.6)	
Triple therapy (%)	27 (34.6)	25 (51.0)	2 (6.9)	
Invasive CTEPH treatment		–		–
BPA (%)	12 (42.9)		12 (42.9)	
PEA (%)	6 (23.1)		6 (23.1)	
Diuretic use (%)	37 (47.4)	20 (40.8)	17 (58.6)	0.099
Other medication use				
Beta blocker (%)	14 (18.2)	8 (16.3)	6 (20.7)	0.760
Calcium channel blocker (%)	9 (11.7)	4 (8.2)	5 (17.2)	0.273
ACE inhibitor/ARB (%)	8 (10.4)	3 (4.1)	5 (17.2)	0.131
Antidepressants (%)	3 (3.9)	2 (4.1)	1 (3.4)	0.702
Oxygen therapy (%)	12 (15.4)	5 (10.2)	7 (24.1)	0.128

*6MWD* 6 min walking distance, *ACE* angiotensin converting enzyme, *ARB* angiotensin receptor blocker, *BMI* body mass index, *BPA* balloon pulmonary angioplasty, *COPD* chronic obstructive pulmonary disease, *ET-1* endothelin-1, *NYHA* New York Heart Association, *P(A)H* pulmonary (arterial) hypertension, *PDE5* phosphodiesterase 5, *PEA* pulmonary endarterectomy

### Problems before and during sexual activity

The results of the SePHia questionnaire are depicted in Table 2. Overall, 30.8% of the patients find it important to discuss sexuality in the doctor’s office. Men found this more important than women (46.2% vs 23.1%; *p* = 0.032). Strikingly, from the patients who are in a committed relationship, 69.0% of patients would like to discuss sexuality with their healthcare providers. Patients did not demonstrate a clear preference with whom they want to discuss sexuality (doctor, nurse specialist or sexologist). The majority of the patients experienced cardiopulmonary complaints during sexual activities. The most frequent symptom was dyspnea (52.6%), followed by palpitations (32.1%) and chest pain (10.3%). In 43.3% of the patients, these symptoms resulted in less sexual activity. Sexual interest was affected by the patients physical and mental condition in respectively 44.9% and 23.1% of the cases. Intravenous or subcutaneous pump therapy affected sexuality in respectively 17.6% and 8.3% of patients.

**Table 2:** Results of the SePHia questionnaire in the overall population and for men and women separately

	Total	Women	Men	*p* value
Do you consider it important to discuss sexuality in the doctor’s office?, yes (%)	24 (30.8)	12 (23.1)	12 (46.2)	0.037
If yes, with whom do you prefer to discuss sexuality?				0.441
Doctor (%)	12 (50.0)	6 (50.0)	6 (50.0)	
Nurse specialist (%)	13 (54.2)	7 (58.3)	6 (50.0)	
Sexuologist (%)	11 (45.8)	5 (41.7)	6 (50.0)	
Does your physical condition as a consequence of PH affect your interest in sex?, yes (%)	35 (44.9)	26 (51.0)	9 (34.6)	0.173
Does your mental condition as a consequence of PH affect your interest in sex?, yes (%)	18 (23.1)	12 (23.5)	6 (23.1)	0.965
Do you experience the following symptoms during sex?				
Dyspnea (%)	41 (52.6)	25 (50.0)	16 (64.0)	0.251
Palpitations (%)	25 (32.1)	19 (38.0)	6 (24.0)	0.225
Chest pain (%)	8 (10.3)	5 (10.0)	3 (12.0)	0.791
Does the presence of symptoms result in less sex?, yes (%)	26 (43.3)	18 (45.0)	8 (40.0)	0.868
**For patients receiving pump therapy**				
Does the presence of interavenous pump therapy negatively affect your sexual activities?, yes (%)	3 (17.6)	2 (20.0)	1 (14.3)	0.761
Does the presence of subcutaneous pump therapy negatively affect your sexual activities?, yes (%)	1 (8.3)	1 (14.3)	–	–

### Sexual health related quality of life in women

**Fig. 2:**
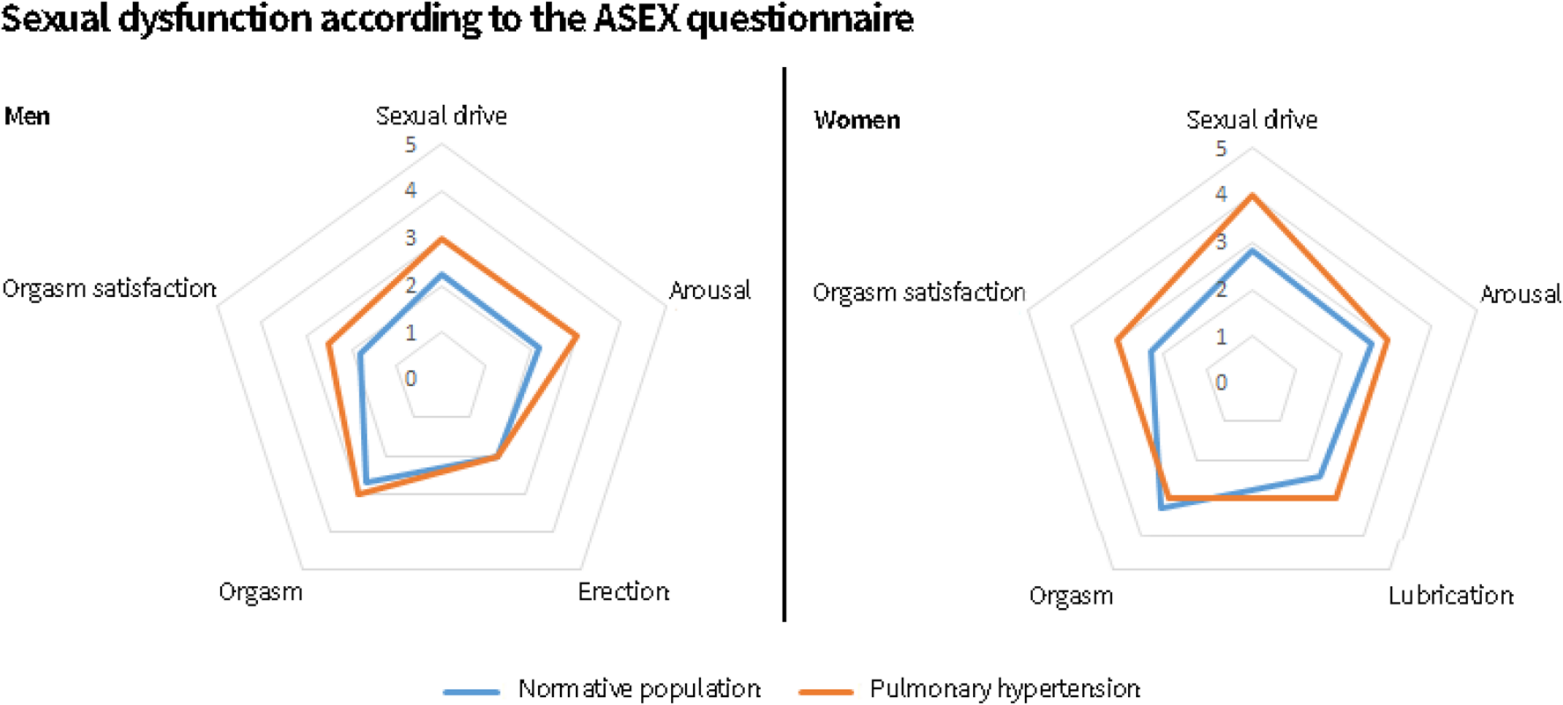
Radar plot comparing the difference in Arizona Sexual Experience Score (ASEX) between the normative population (blue) and patients diagnosed with pulmonary hypertension (orange) for men (left) and women (right). High scores indicate worse sexual health related quality of life

**Fig. 3:**
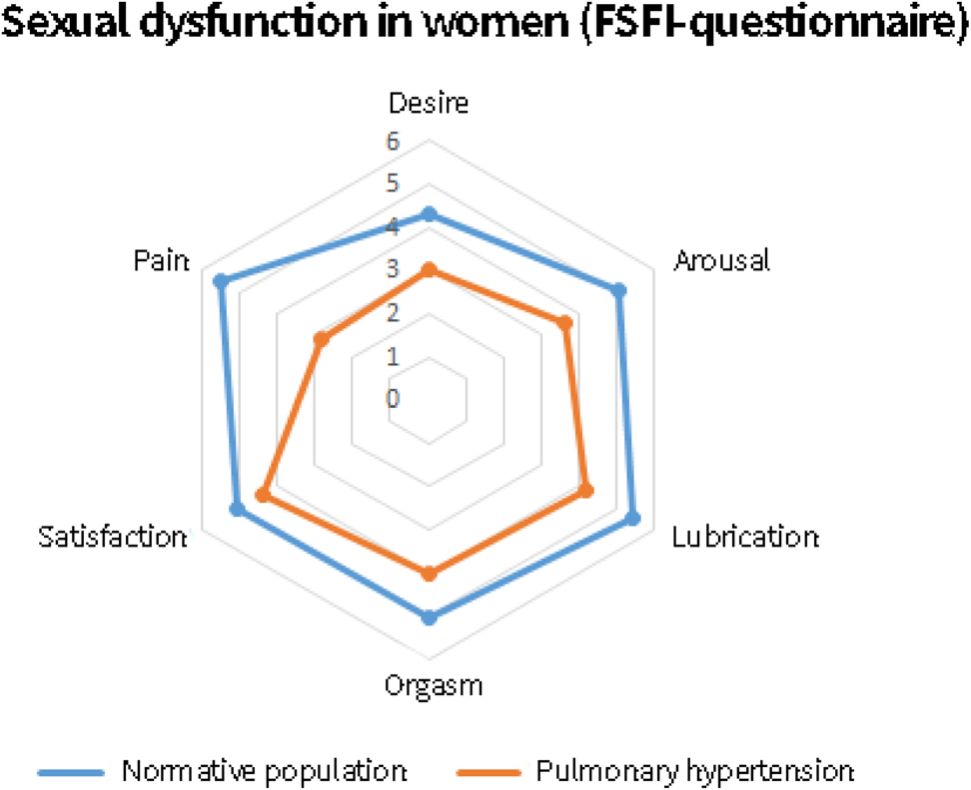
Radar plot comparing the difference in Female Sexual Function Index (FSFI) score between the normative population (blue) and women diagnosed with pulmonary hypertension (orange). Low scores indicate worse sexual health related quality of life

Women with PH score significantly worse on arousal, sexual drive, lubrication and orgasm satisfaction compared to the normative population based on both the ASEX and FSFI questionnaires (Figs. 2 and 3). Based on the FSFI women with PH score worse on orgasm and pain scales, this highlights the broad range of sexual problems that women with PH face.

In the general Dutch population of women aged 55–69 years, sexual dysfunction is present in 19% of the women [28]. Sexual dysfunction was present in 63.0% of the women with PH based on the FSFI questionnaire and in 42.2% based on the combined scores of the FSFI and FSDS questionnaires.

### Sexual health related quality of life in men

Sexual functioning is severely impaired in men diagnosed with PH and effects multiple domains of sexuality according to the ASEX questionnaire. Figure 2 shows that arousal, sexual drive, orgasmic function and orgasm satisfaction were significantly worse compared to the norm population.

**Fig. 4:**
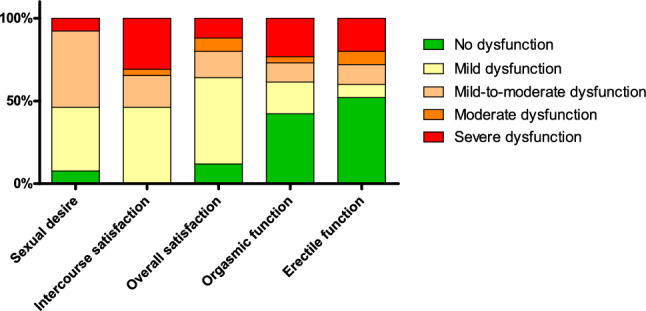
Presence of sexual dysfunction in men per domain according to the International Index of Erectile Function (IIEF) questionnaire

In the general Dutch population, sexual dysfunction is present in at least one domain in 11% of men aged 55–69 years [28]. In our study population according to the IIEF, 92.3% of the men diagnosed with PH experienced at least mild dysfunction in sexual desire and all men experienced decreased intercourse satisfaction (Fig. 4). Only 12.0% of men experienced no dysfunction in overall satisfaction and 42.3% had preserved orgasmic function. Erectile dysfunction was present in 48.0% of the patients.

### Disease related factors associated with sexual dysfunction

**Fig. 5:**
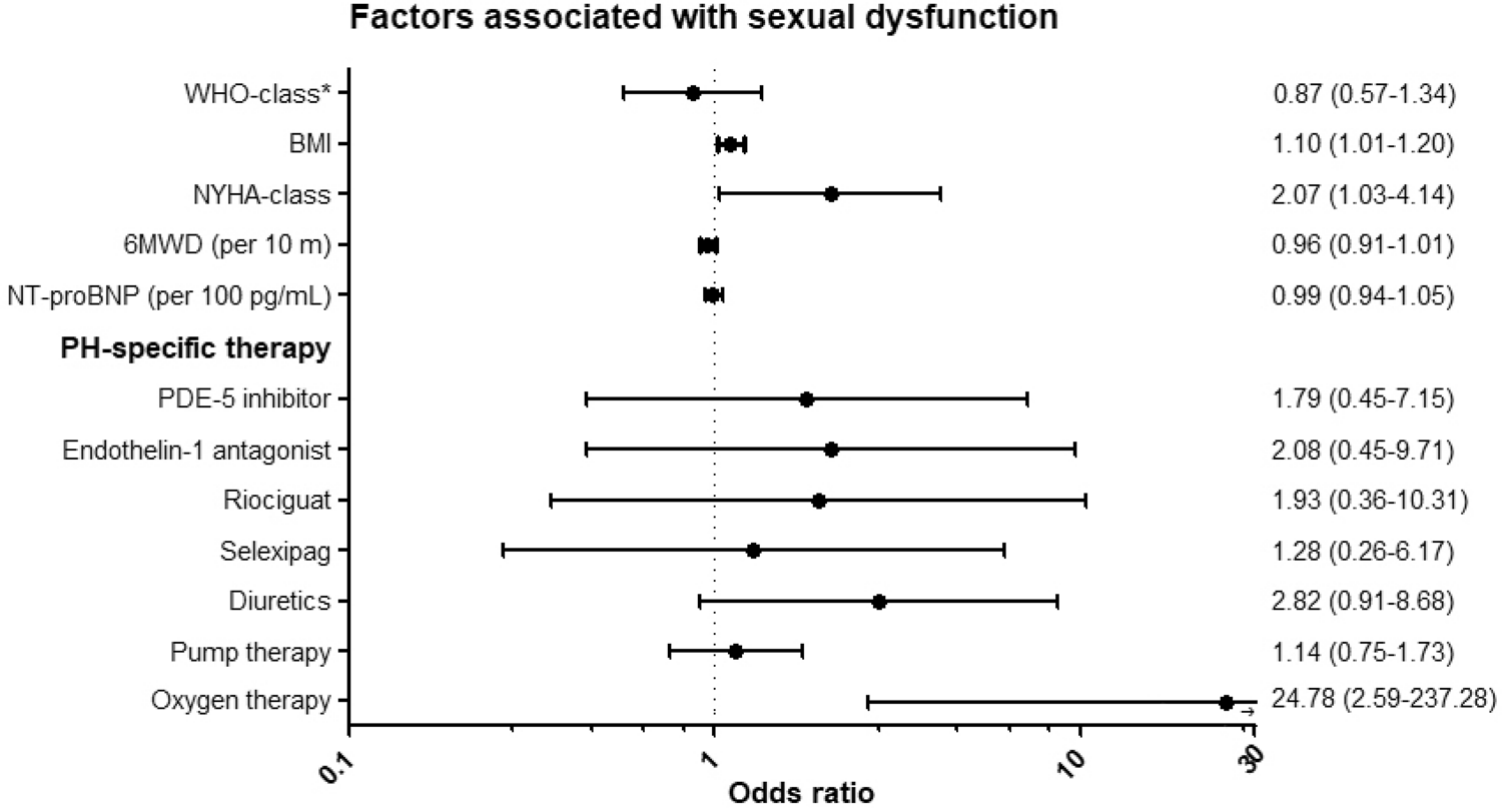
Risk of sexual dysfunction for clinical characteristics and PH-specific therapy, adjusted for age and sex. *Reference: pulmonary arterial hypertension. *6MWD* 6 min-walking distance, *BMI* body mass index, *NYHA* New York Heart Association, *PDE-5* phosphodiesterase-5

Based on the ASEX questionnaire, sexual dysfunction is present in 25 patients (32.1%) of the total population. Factors associated with sexual dysfunction were identified using multivariable logistic regression adjusting for age and sex (Fig. 5). There was no difference in sexual dysfunction between different diagnostic groups. A significant association with sexual dysfunction was found for BMI (OR 1.10, 95%-CI: 1.01–1.20) and NYHA-class (OR 2.07, 95%-CI [1.03–4.14]). The use of PH-specific therapy, more specifically the use of PDE-5 inhibitors, endothelin-1 antagonist, riociguat and selexipag was not associated with sexual dysfunction. Furthermore, receiving pump therapy did not affect sexual functioning in our population (OR 1.14, 95%-CI: 0.75–1.73). Patients who need oxygen therapy show a significantly higher risk for sexual dysfunction. The use of diuretics showed no association with sexual dysfunction in the overall population (OR 2.82, 95%-CI: 0.91–8.68). However, when only women were analyzed, a significant association was found between diuretic use and sexual dysfunction (OR 4.01, 95%-CI: 1.04–15.41). Besides diuretic use, no other sex-related differences were observed.

## Discussion

This study demonstrated severely impaired sexual functioning in both women and men with pulmonary hypertension compared to the general population. More than half of the included patients reported worsening of symptoms during sexual activity, with dyspnea being the most prominent. Up to two out of three patients consider sexuality an important issue that should be addressed during follow-up. Both men and women experienced a broad range of sexual problems, including lower sexual drive, arousal, orgasm, orgasm satisfaction and impaired lubrication or erectile function.

Patients with chronic diseases are often confronted with sexual dysfunction [31]. Factors that play a role in this include symptoms, disabilities, low self-esteem and negative body image due to the disease and visible scars or devices, worsening of symptoms and fear of pregnancy. In respiratory medicine, sexual dysfunction was found in patients suffering from a broad range of diseases from asthma to lung cancer [32, 33]. In patients with COPD, sexual dysfunction was highly prevalent and negatively impacted HRQoL [34]. The same was demonstrated in PH as lower SHRQoL is associated with worse HRQoL [23].

Patients with PH are confronted with a number of factors that influence their biopsychosocial wellbeing and thus sexuality. Deterioration of cardiopulmonary symptoms during sexual activities was reported by the majority of patients. The discomfort of these complaints as well as the fear for worsening of symptoms or possible complications could lead to decreased interest in sex and sexual satisfaction. Intercourse and orgasm satisfaction are significantly lower in women with PH compared to the normative population and all men in our cohort report decreased intercourse satisfaction.

Research on sexuality in PH is scarce, and sexuality has only been described in women with PAH so far. One study showed a prevalence of 71.8% for sexual dysfunction in women with PAH based on the FSFI questionnaire [22]. In this study, we found sexual dysfunction in 63.0% of the women based on the FSFI. Using the combined score of the FSFI and the FSDS resulted in a lower prevalence of sexual dysfunction (42.2%). The FSDS is a measurement of sexual distress. A possible explanation for the difference in sexual dysfunction between the FSFI and the combined FSFI & FSDS is that despite the presence of impaired sexual function, some patients may not be distressed about this.

All male patients reported sexual dysfunction in at least one domain. This is higher than for example in patients with COPD [34]. A possible explanation for this could be that respiratory symptoms might be more severe in patients with PH and that PH patients are relatively younger at the time of diagnosis. Erectile dysfunction has a high prevalence of 42–75% in patients with cardiac disease [35]. This is similar to the 45.5% we found in our PAH and CTEPH population. However, in our population erectile dysfunction might even be underestimated as the majority of patients is treated with a PDE-5 inhibitor which improves erectile function.

We found no difference in SHRQoL between patients with PAH and CTEPH. Higher BMI and NYHA-class were associated with sexual dysfunction. Individual PH-specific oral medication did not affect sexuality. Banerjee et al. did find worse FSDS scores in women treated with intravenous or subcutaneous prostanoids [23]. Our study could not reproduce these findings as intravenous or subcutaneous therapy was not associated with sexual dysfunction. Diuretics were not associated with sexual dysfunction in men, but they were associated with sexual dysfunction in women. This could be due to impaired lubrication as a side-effect. Oxygen therapy was associated with a severe increase in sexual dysfunction, possibly due to nasal cannulas that can pose difficulties, but it also reflects more severe underlying disease.

Despite the high prevalence of disease-related sexual dysfunction, sexuality is often not discussed by healthcare professionals or patients. It is known that both patients and healthcare providers find it difficult to talk about sexuality [36]. In our questionnaire, especially patients who are in a committed relationship expressed the need to discuss sexuality with their healthcare providers. This emphasizes the need of patients to discuss sexuality and healthcare providers should take initiative in the screening for sexual dysfunction. In a survey among Dutch cardiologists, only 16% stated to discuss sexuality with their patients. Of all patients, only 2% was referred for help with a sexual problem [37]. In a survey among patients with chronic coronary artery disease, only 3% of men and 18% of the women thought they were adequately informed about sexuality and their disease. It however also reports that sexual dysfunction was more likely to be discussed with men than with women [38]. Sexual health remains a taboo in healthcare, while the problem among patients with PH is real. Patients, especially those who are in a committed relationship, do express the wish to discuss sexuality in function of their disease. One of the reasons that doctors don’t discuss sexuality with their patients could be that they think that there are no interventions for sexual dysfunction. However, sexological healthcare was found to be beneficial in patients with chronic diseases [39]. Furthermore, doctors can provide adequate information and take away fears that patients could deal with that withholds them from engaging in sexual activities. Healthcare providers should take initiative in this and have to proactively discuss sexuality with their patients to provide them with adequate information regarding sexuality and refer them to a sexologist if needed [40].

### Strengths and limitations

This is one of the only studies investigating SHRQoL in patients with PH and, to our knowledge, the first to investigate sexual dysfunction in men. We used semi-structured interviews to extract PH-specific difficulties with sexuality. SHRQoL was assessed using multiple validated questionnaires. The SePHia-questionnaire was constructed to evaluate specific problems based on the semi-structured interviews. There are also some limitations. In our study population, a relatively large proportion of patients is in NYHA-class I or II, sexual dysfunction might therefore be underestimated. We were not able to perform stratified analysis for different PAH etiologies or due to limited sample size able to correct for other factors than age and sex such as beta blocker use. This study was performed in patients with PAH and CTEPH; it might therefore not be completely generalizable to other types of PH.

## Conclusion

The majority of men and women with PAH and CTEPH experience symptoms during sexual activities, the most prevalent symptom being dyspnea. Up to 69% of patients want to discuss sexuality with their healthcare provider. Sexual dysfunction and distress are present in more than half of the patients. It is therefore important for doctors and (specialized) nurses to discuss sexuality with patients to adequately inform the patients and refer them for specialized sexological healthcare if necessary.

## Data Availability

The data are available from the corresponding author on reasonable request.
